# Shape-based disease grading via functional maps and graph convolutional networks with application to Alzheimer’s disease

**DOI:** 10.1186/s12880-024-01513-z

**Published:** 2024-12-18

**Authors:** Julius Mayer, Daniel Baum, Felix Ambellan, Christoph von Tycowicz, for the Alzheimer’s Disease Neuroimaging Initiative

**Affiliations:** Visual and Data-centric Computing, Zuse Institute Berlin, Takustraße 7, Berlin, 14195 Berlin Germany

**Keywords:** Shape analysis, Computer-aided diagnosis, Geometric deep learning, Alzheimer’s disease

## Abstract

Shape analysis provides methods for understanding anatomical structures extracted from medical images. However, the underlying notions of shape spaces that are frequently employed come with strict assumptions prohibiting the analysis of incomplete and/or topologically varying shapes. This work aims to alleviate these limitations by adapting the concept of *functional maps*. Further, we present a graph-based learning approach for morphometric classification of disease states that uses novel shape descriptors based on this concept. We demonstrate the performance of the derived classifier on the open-access ADNI database differentiating normal controls and subjects with Alzheimer’s disease. Notably, the experiments show that our approach can improve over state-of-the-art from geometric deep learning.

## Introduction

Shapes of anatomies and variations thereof pose a key source for the understanding of medical phenomena including physiological processes like tissue malformations associated to pathological conditions. This, in turn, plays a significant role in medical decision-making, e.g., stratification for clinical interventions or therapy planning.

From a mathematical point of view, shapes are an instance of geometric data that requires dedicated computational treatment. There is increasing evidence that data-analytical tools that account for the inherent geometric structure yield improved consistency and performance, see [1] and the references therein. This led to a strong impetus to generalize established geometric approaches that have been derived for Euclidean data, affecting deep learning and statistics, as well as data processing and visualization.

Central to morphological analysis is the comparison of related forms. This requires a coordinization of shapes leading to a notion of shape space in which each point represents a specific shape [2]. An established approach is to consider transformations connecting shapes: An object collection under study can be represented by a common deformable template that accounts for the characteristics of the structure within the collection. The shape variability is then represented by deformations that are applied to the template. Within this approach, Riemannian methods have shown promising results for tasks such as the discovery of biomarkers [3], risk assessment of clinical outcomes [4], and longitudinal analysis [5, 6].

Despite these advances, frameworks for geometric morphometry still rely on point-to-point correspondences between shapes, either explicitly in form of homologous landmarks [7] or implicitly in terms of diffeomorphisms of the ambient space [8]. Point-to-point correspondences have fundamental limitations that impede the analysis of shape collections with topologically varying or incomplete objects. This is a major problem for the analysis of empirically given sets of shapes since they, due to either real differences or reconstruction errors, often contain such objects. Even though this issue is extremely common, it is frequently disregarded or viewed as unavoidable, potentially introducing bias into subsequent analyses [9].

Therefore, novel concepts that pose less strict assumptions are of high interest. While recent work on non-rigid registration allows for partial matching [10] and topological changes via user-specified [11] or unsupervised detection [9] of discontinuities, extensions to group-wise analysis are still at an early stage of research. A promising alternative approach [12] that we evaluate in this work is to generalize the notion of correspondence between shapes in terms of maps between real-valued functions on the surfaces instead of points thereon. Remarkably, such *functional maps* facilitate shape matching [13] and still allow for a notion of shape differences that characterize distortion between shapes [14]. Furthermore, recent advances for improving cycle consistency in functional map networks [15, 16] via latent representations give rise to novel types of shape spaces, the full potential of which remains to be explored.

Another alternative approach to explicit shape spaces is to infer the underlying structure entirely from the data at hand. Deep learning has led to qualitative breakthroughs for various tasks there [17–19]. As shapes are described by curved surfaces, they are geometric objects in their own right and require dedicated neural network units. The study of such units falls into the field of geometric deep learning. We refer to [20] for an overview. Beyond architectures for digital surfaces, geometric deep learning provides graph neural networks for data featuring irregular, heterogeneous relationships. In this area, graph convolutional networks (GCN) have been shown to provide effective transductive learning schemes for disease classification from imaging [21] as well as shape-based features [22].

### Contributions

Following the transductive learning design, we derive a classification approach that casts the grading task as a semi-supervised node classification problem on a shape-valued graph. To this end, we adapt a flexible, yet descriptive characterization of shape variability based on the framework of functional maps that poses no assumptions on the existence of underlying diffeomorphic correspondences. Furthermore, we generalize functional shape characterizations introducing a one-parameter family of descriptors that provide a tuneable sensitivity to extrinsic curvature.

We evaluate the performance of the proposed classifier considering hippocampus malformations caused by Alzheimer’s disease and achieve state-of-the-art accuracies outperforming recent approaches based on functional maps [16] as well as geometric deep learning [17]. This work is an extended version of the workshop paper [23] featuring a more comprehensive experimental evaluation, an extended data set containing both left and right hippocampi, and additional visualizations. Based on the new findings, we derive an improved classifier for Alzheimer’s disease from hippocampi shapes that surpasses our previous design by a significant margin.

## Shape analysis

In this section, after giving some background and setting the notation, we introduce the novel shape descriptors and show that they are suitable to recognize changes in extrinsic geometry within a shape collection.

**Fig. 1:**
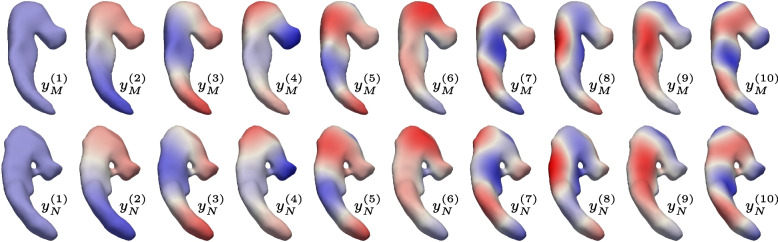
A consistent latent basis on two hippocampal shapes of different genus. $-1$  1

### Functional maps

Let $\mathcal {S}$ be a set of manifolds and $S\in \mathcal {S}$. Let $X_S$ be a space of $\mathbb {R}$-valued functions on *S* endowed with some basis. Then, [12] defines functional correspondence between $M,N\in \mathcal {S}$ as an operator $F_{M,N}:X_M\rightarrow X_N$ named functional map. It can be encoded by a matrix $C_{M,N}$ such that $a_N\approx C_{M,N}a_M$ for any function in $X_M$ with coordinates $a_M$ corresponding to a function in $X_N$ with coordinates $a_N$. Let $T:N\rightarrow M$ be a point correspondence map. If $X_S$ is chosen to be the set of all $\mathbb {R}$-valued functions on *S*, then the functional map ${F:X_M\rightarrow X_N, f\mapsto f\circ T}$ comprises the same information as *T*.

### Consistent latent basis

Let $(\mathcal {S}, E)$ be a connected directed graph with vertices $\mathcal {S}$ and edges $E\subset \mathcal {S}\times \mathcal {S}$. For each $(M,N)\in E$, let there be a functional map. Let $Y_S\subset X_S$ and $F_{M,N}(Y_M)=Y_{N}$. Then, following [15], we call $(Y_S)_{S\in \mathcal {S}}$ the latent space of $(X_S)_{S\in \mathcal {S}}$.

Let $(y_S^{(i)})_{i\in I}$ be a basis of $Y_S$ such that

$$\begin{aligned} F_{M,N}y_M^{(i)}=y_N^{(i)}\quad \forall \,M,N\in \mathcal {S},\,i\in I. \end{aligned}$$

Then, $(y_S^{(i)})_{S\in \mathcal {S},i\in I}$ is called consistent latent basis (CLB) of $(Y_S)_{S\in \mathcal {S}}$. Figure 1 shows an example of a latent basis for two hippocampal shapes. Note that such a basis can be constructed even if topological variation is present in the shape collection.

### Shape difference descriptors

**Fig. 2:**
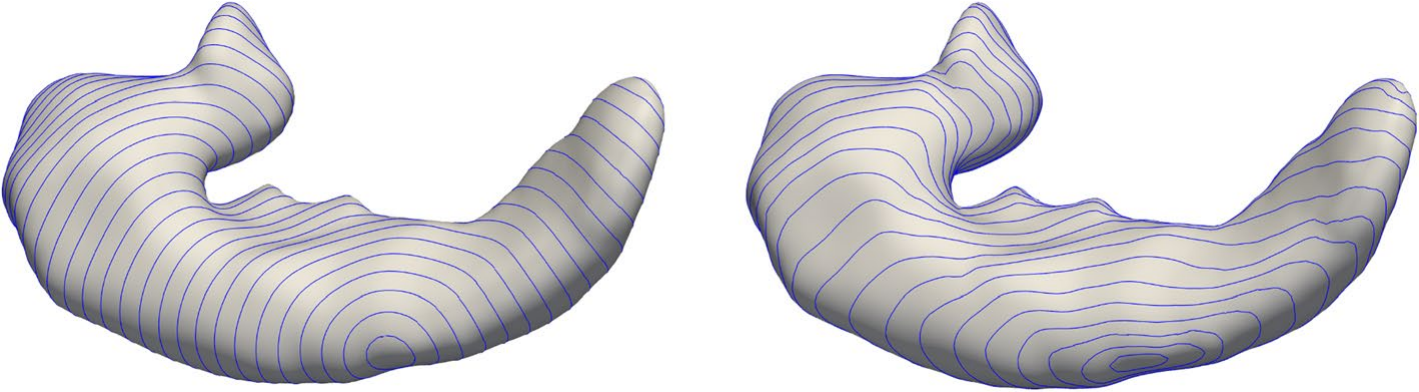
Comparison of distance isolines. The standard metric (left) does not take extrinsic geometry into account, in contrast to the isophotic metric (right, with $\omega$ = 8)

Let *S* be a regular orientable surface [24], $\textrm{I}_S$ denote its first and $\textrm{III}_S$ its third fundamental form. For $\omega \ge 0$, let $g_{S,\omega }:=\textrm{I}_S+\omega \textrm{III}_S$ be the regularized isophotic metric [25] on *S* and $\mu _{S,\omega }$ denote its Riemannian density [26]. Figure 2 shows distance functions for a hippocampus surface equipped with the induced metric $\textrm{I}_S$ as well as the isophotic metric $g_{S,\omega }$ illustrating the dependence of the latter on the variation of the surface normals along connecting geodesics.

Let $L^2(S,\omega )$ denote the set of square integrable real-valued functions on $(S,g_{S,\omega })$ and let $H_{\emptyset }^1(S,\omega ):=\{f:\,D^{\alpha }f\in L^2(S,\omega )\;\forall \,|\alpha |\le 1, \int _Sfd\mu _{S,\omega }=0\}$ where $D^{\alpha }$ denotes the weak $\alpha$-th partial derivative using multi-index notation. Let

$$\begin{aligned} h_{S,\omega }^a & : L^2(S,\omega )\,\times L^2(S,\omega )\,\rightarrow \mathbb {R},(f_1,f_2)\mapsto \int _S f_1f_2\;d\mu _{S,\omega }\\ h_{S,\omega }^c & : H^1_{\emptyset }(S,\omega )\times H^1_{\emptyset }(S,\omega )\rightarrow \mathbb {R}, (f_1,f_2)\mapsto \int _S \nabla f_1^T\nabla f_2\;d\mu _{S,\omega } \end{aligned}$$

where $\nabla$ is the del operator in terms of weak derivatives. Then, [27] shows

$$\begin{aligned} h_{M,0}^a(f_1,f_2)=h_{N,0}^a(F(f_1),F(f_2))\,\forall \,f_1,f_2\in L^2(M,0)\;\, & \Leftrightarrow \,T\text { is locally area-preserving,}\\ h_{M,0}^c(f_1,f_2)=h_{N,0}^c(F(f_1),F(f_2))\,\forall \,f_1,f_2\in H^1_{\emptyset }(M,0)\, & \Leftrightarrow \,T\text { is conformal.} \end{aligned}$$

If *T* is locally area-preserving and conformal, then it is an isometry, i.e., it preserves intrinsic geometry. This motivates to compare inner products of corresponding functions, e.g., CLBs, to capture shape differences.

**Fig. 3:**
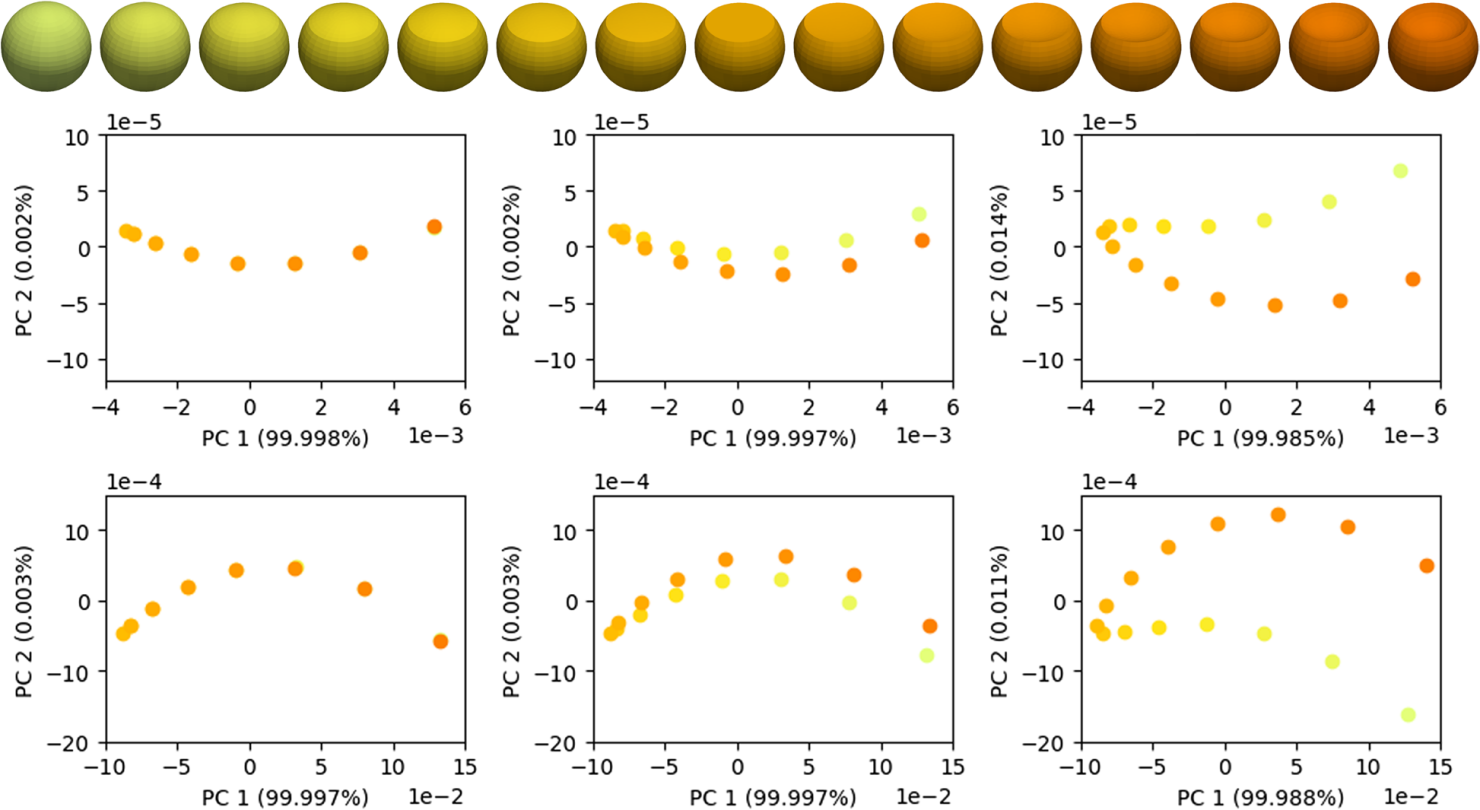
PCA plot of $h^a_{S,\omega }$ (top) and $h^c_{S,\omega }$ (bottom) for $\omega =0$ (left), $\omega =0.005$ (middle) and $\omega =0.01$ (right) where the color of the shapes matches the color of the points in the plots

For $\omega>0$, the inner products $h_{S,\omega }^a$ and $h_{S,\omega }^c$ also contain information about the extrinsic geometry. Different to [14], this approach does not require to construct an offset surface. Figure 3 shows that the parameter $\omega$ actually allows to control the influence of the extrinsic geometry. It depicts linear interpolations $S_t$ between a sphere $S_0$ and a shape $S_1$ generated by mirroring the upper dome of the sphere at the cutting plane. Consequently, the shape $S_{^{1}\!/_{2}}$ in the middle of the series is flat at the top and shapes $S_t, S_{1-t}$ are pairwise isometric.

For the purely intrinsic shape differences $h_{S,0}^a$ and $h_{S,0}^c$, these isometric pairs are indistinguishable, while the disparity grows with increasing $\omega$, so that we can observe an unfolding of the series in the PCA plot.

## Disease classification

As an example for disease grading, we classify states of Morbus Alzheimer also known as Alzheimer’s disease (AD), for which we derive a neural classifier. AD is a rapidly progressing and most prevalent neurodegenerative disorder among the elderly and is a primary cause of dementia. AD causes considerable distress in patients, hindering their capacity to carry out basic everyday activities and ultimately leading to death. In the following, after providing details on the data used and how we processed them, we explain the architecture of the proposed network.

### Data

Morbus Alzheimer is known to affect large regions of the human brain [28] such as the two hippocampi [29], which play an important role in the formation of memories [30]. In order to take into account morphological changes, we examine the hippocampi shapes represented as triangle meshes that we derived from MRI scans published by the Alzheimer’s Disease Neuroimaging Initiative (ADNI)^1^. ADNI is a longitudinal, multi-center, observational study designed to characterize the trajectories of clinical, imaging, and fluid biomarkers throughout the spectrum of aging from clinically normal individuals through mild cognitive impairment to AD, with data made available publicly. The goal is to identify biomarkers and genetic characteristics that would support the early detection and follow-up of AD, as well as the improvement of the design of clinical trials. ADNI was initially funded in 2004 and recruited 819 people in this phase (ADNI-1) and was further extended in 2009 (ADNI-GO) and 2010 (ADNI-2) by 129 and 782 participants, respectively. A detailed description of the study design and participants can be found in [31]; for up-to-date information, see www.adni-info.org. All phases of the study collected clinical data (neuropsychological testing, neurological examination and diagnosis) and sMRI in all participants. In particular, from the available phenotypic data, we collected information on sex and Apolipoprotein E (ApoE) gene for the respective subjects.

We carry out our experiments on the data set derived in [32] consisting of 120 subjects with only shapes of right hippocampi and a new, larger data set of 238 subjects with shapes of left and right hippocampi. The first data set comprises 60 subjects with Alzheimer’s disease and 60 cognitive normal controls. All of their hippocampi shapes have genus 0 and therefore show no topological variation. The second data set consists of all available baseline observations in ADNI-2 comprising 101 diseased and 137 normals, where one left and one right shape of each the diseased as well as the normals have genus 1 instead of genus 0. Note that the higher-genus instances are due to imaging/segmentation artifacts such as partial voluming. Such cases do not admit diffeomorphic matching, and hence need to be excluded in shape analysis approaches based on point-to-point correspondences unless topology is fixed in a possibly expensive preprocessing step. In contrast, functional correspondences alleviate these limitations, allowing inclusion of all instances in the analysis. Examples of both topological cases can be seen in Fig. 1.

### Classification

Typically, the sampling in clinical data sets does not follow a regular grid, and there exists no natural ordering. However, individuals feature heterogeneous pairwise relationships and interdependencies that can be adequately captured by a graph. In this setting, nodes represent subject-specific shapes, while edge weights can be used to encode similarities between subjects potentially integrating auxiliary, phenotypic information.

**Fig. 4:**
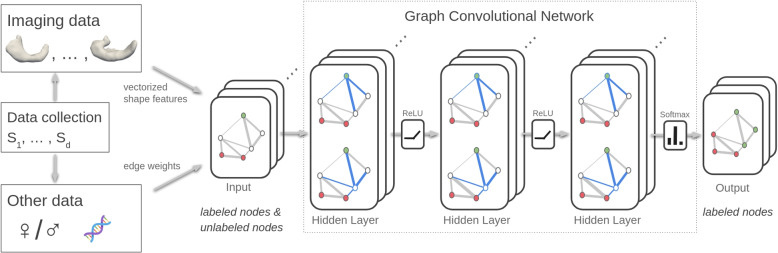
Illustration of the semi-supervised node classification we propose for disease grading

Based on the graph representation, the transductive inference problem can be formulated as semi-supervised node classification, where labels are only given for nodes corresponding to subjects from the training set. As classifier, we construct a multi-layer, feed-forward graph convolutional network with possibly several hidden layers each followed by a rectified linear unit (ReLU), see Fig. 4.

The final layer has as many output channels as the desired number of classes and is equipped with a node-wise soft-max activation. As loss function, a cross-entropy term for each node in the training set is used. Since the model is conditioned on the adjacency of the graph, there is no need for explicit graph-based regularization: The gradient information of the loss is propagated through the model enabling it to learn representations of both labeled and unlabeled nodes. For our architecture, we opt for a spectral generalization of graph convolutions, which are based on a $K^{th}$-order approximation in terms of Chebyshev polynomials [33] and provide fast localized graph convolutions with constant learning complexity.

While the construction of the functional map network is driven by geometric considerations (viz. establishing consistent, group-wise correspondences), it does not encode phenotypic relationships and interdependencies between the subjects that are informative to the grading task. We therefore condition our model on another graph that leverages both geometric and non-geometric information. Following [21], we define the adjacency matrix *W* of the population graph by

$$\begin{aligned} W_{ij} = Sim(S_i, S_j) \sum \limits _k \delta (m^k_i, m^k_j), \end{aligned}$$

where *Sim* gauges similarity between subject shapes and $\delta$ is a threshold function testing for closeness of phenotypic measures $m^k$ such as sex and ApoE type. Whereas the exact choice of *Sim* should be application-dependent, a canonical candidate is to employ a radial basis function kernel based on distances of the node features.

## Processing

Let $\mathcal {S}$ contain shapes of only left or only right hippocampi. We choose $X_S$ to be the space spanned by the eigenvectors of the Laplace-Beltrami operator associated to its smallest 42 eigenvalues, where we discretize the operator using the standard weighted cotangent scheme [34]. For a dimension higher than 42, we did not observe improvements in the classification accuracy. Then, we compute $C_{M,N}$ by using ZoomOut refinement [13]. As initialization, we take a $30\times 30$ matrix that encodes correspondences of 130 approximate landmarks obtained via coherent point drift and perform 12 steps that each increase the dimension of the spaces $X_S$ by 1. As these functional maps only need to capture the correspondence approximately, we chose the parameters to result in small deformations between each pair of shapes in terms of the Green-Lagrangian strain tensor.

To avoid considering bad correspondence maps, we define the edges *E* of the functional map network by the (symmetrized) *k*-nearest-neighbor graph based on the 2-cycle consistency given by $||C_{N,M}C_{M,N}-I||$ where *k* is the smallest value such that $(\mathcal {S},E)$ is actually connected. Then, we compute a CLB of $(F_{e})_{e\in E}$ by using Consistent ZoomOut [35]. As common parameter choices for spectral upsampling, we initialize it with the leading principal $8\times 8$-submatrices of all $C_{M,N}$ with $(M,N)\in E$ and perform 20 steps that each increase the dimension of $X_S$ by 1 and of $B_S$ by 7/10.

The inner products $h_{S,\omega }^a$ and $h_{S,\omega }^c$ on $Y_S\times Y_S$ can be represented by symmetric positive-definite (SPD) matrices. To account for the geometric structure of the space of SPD matrices, we employ the Log-Euclidean framework [36]. To this end, we consider the matrix logarithms of the SPD matrices. For use as network features, we build a vector out of the entries of each logarithm and divide the vectors by their respective 2-norm to regularize them. Since the matrix logarithm of a SPD matrix is still symmetric, we only take the entries of the respective lower-triangles to avoid redundant information.

We evaluate the proposed graph convolutional network (GCN) for the discrimination between normal controls and subjects with Alzheimer’s disease. To this end, we employ three layers of second-order graph convolutions with input dimensions ($n_{\textrm{shapes}}, 64, 64$). As phenotype measures, we selected sex and ApoE genotype following the work [21]. Gene ApoE appears in three major types: E2, E3 and E4. Especially E4 is known as genetic risk factor for Alzheimer’s disease. Let $d_{ij}$ be the distance between the shape difference descriptors of $S_i$ and $S_j$ in terms of the Log-Euclidean framework. We then define $Sim(S_i,S_j)=\text {exp}(-0.5d_{ij}^2/\sigma ^2)$, where $\sigma$ is the median of the distances of all shape pairings. If both sex and ApoE type coincide and one of the shapes, $S_i$ or $S_j$, is among the 30 closest neighbors of the other, we set the threshold to 1. Otherwise, we set it to 0.

## Results

On two Intel^®^Core$^{\text {TM}}$ i9-10920X, the computation time for the shape correspondence in terms of a CLB amounts to 15 hours and 24 minutes for one side of the hippocampi. This could probably be reduced by taking matrices of lower dimension for ZoomOut without compromising the quality of the CLB. On the same processor, the classification task considering both sides of the hippocampi took 12 minutes for the set of 120 subjects and 39 minutes for the set of 238 subjects for each choice of $\omega$.

### Comparison to state-of-the-art

**Fig. 5:**
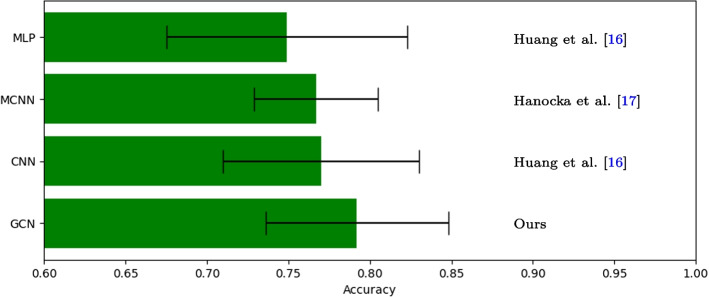
The proposed GCN approach achieves the highest average classification accuracy of $0.787 \pm 0.055$ followed by CNN ($0.77 \pm 0.06$), MCNN ($0.767 \pm 0.038$) and MLP ($0.749 \pm 0.074$) [16, 17]

We evaluate the performance of our approach in terms of classification accuracy in comparison to state-of-the-art approaches for shape-based learning that do not require point-to-point correspondences. On the one hand, we employ classification approaches using a MultiLayer Perceptron ($\textrm{MLP}$), as well as a Convolutional Neural Network ($\textrm{CNN}$) applied on the area- and conformal-based shape differences as proposed in [16], however, using our generalized differences based on the isophotic metric. On the other hand, we show results for MeshCNN [17] ($\textrm{MCNN}$) as reported by [32] (note that these are rather an upper bound for the accuracy since maximal test accuracies are reported instead of using proper stopping criteria). In contrast to first methods based on the functional maps framework, MCNN is applied directly on the shape-forming triangular mesh. Note that MLP, CNN and MCNN are following the *inductive* learning approach. We evaluated all methods on a $70\%/30\%$ training/testing split, performing a stratified Monte Carlo cross-validation drawing 300 times for MLP, CNN, GCN and due to its high computational cost 10 times for MCNN. This comparison employs the data set derived in [32] (see “Data” section). The results, summarized in Fig. 5, indicate that the transductive GCN approach achieves the highest ($79.2\%$) average classification accuracy, CNN and MCNN are approximately on par ($77.0\%/76.7\%$), and MLP achieves the lowest performance with $74.9\%$ accuracy.

### Ablation and hyper-parameter study

We further investigate the performance of the proposed grading system under varying hyper-parameter choices and sets of input anatomies/features. In this set of experiments, we use the extended ADNI data set that not only features a higher cardinality but also comprises shapes of both right and left hippocampi.

**Fig. 6:**
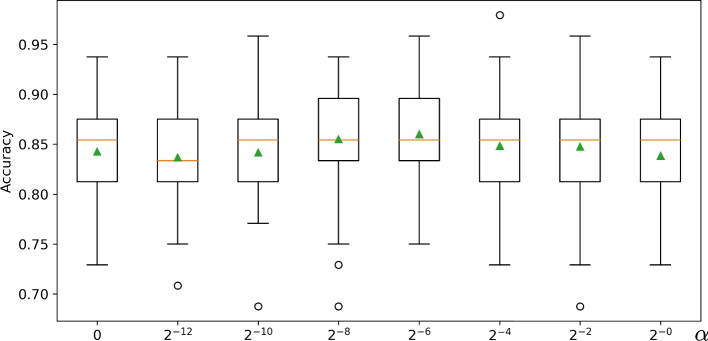
Classification accuracy depending on parameter $\omega$ (in terms of multiples $\alpha$ of the squared length of the bounding box diagonal); higher values yield shape differences with increasing influence of extrinsic geometry, while 0 corresponds to the purely intrinsic differences. (  median;  mean)

We start with an analysis of the classification performance depending on the choice of $\omega$, which weights the contribution of the third fundamental form $\textrm{III}_S$ and, hence, the degree to which the resulting shape differences are aware of the extrinsic geometry of the surface *S*. While $\textrm{III}_S$ is invariant when resizing a surface (e.g. when changing units), the first fundamental form $\textrm{I}_S$ scales quadratically. This motivates to parameterize $\omega$ as a linear combination of a factor $\alpha$ and a surface quantity that also scales quadratically with surface size. In this study, we opted for the simple choice $\omega = \alpha \ell ^2$, where $\ell$ is the length of the bounding box diagonal. Based on this, we investigated the performance of our classifier conditioned on area-based $h^a_{S,\omega }$ and conformal $h^c_{S,\omega }$ shape differences for left and right hippocampi. We sampled $\alpha$ logarithmically in the intervall [0, 1] and performed 100-fold Monte Carlo cross-validation for each sample. Figure 6 provides a summary of the obtained classification accuracies. These results reveal a dependency of the accuracy with a maximal average accuracy of $86.0\%$ for $\alpha = 2^{-6}$. This is a significant improvement (t-test: $t(198)=2.66, p=.008$) over the purely intrinsic shape differences ($\alpha =0$) with an average accuracy of $84.3\%$, demonstrating the advantage of our novel extrinsic shape differences. We further investigated the performance of the best configuration ($\alpha = 2^{-6}$) in terms of precision, recall/sensitivity, and specificity yielding $86.6\%$, $81.0\%$, and $89.9\%$ on average, respectively. The experiment also reveals decreasing performance gains for larger and smaller values of $\alpha$ substantiating the need for task-specific tuning of the extend to which extrinsic and intrinsic geometry are blended—a feature not present in previous functional map-based characterizations such as those based on offset surfaces [27] that underlie numerous constraints [37], e.g. local curvature.

We further performed an ablation study to analyze the accuracy of the proposed grading system when conditioned on different anatomies and shape differences. In particular, we evaluated configurations that take area-based and/or conformal shape differences for right, left, and both hippocampi shapes as input. In Table 1, we provide average classification accuracies determined via 100-fold Monte Carlo cross-validation for the extended ADNI data set as well as the smaller reference data set from [32], which we augmented by the corresponding left hippocampi. Overall, this study shows that area-based shape difference are most informative to the grading task, while the combination with the conformal ones yields the best results. Additionally, we observe an improved accuracy for grading when taking both left and right hippocampi into account, especially for the larger data set that shows up to $5\%$ increase as compared to grading from a single hippocampus.

**Table 1:** Accuracy of proposed classifier for Alzheimer’s disease when conditioned on left and/or right hippocampi as well as area-based and/or conformal shape differences

#	Right			Left			Both		
	$h^a_{S,\omega }$	$h^c_{S,\omega }$	$h^{*}_{S,\omega }$	$h^a_{S,\omega }$	$h^c_{S,\omega }$	$h^{*}_{S,\omega }$	$h^a_{S,\omega }$	$h^c_{S,\omega }$	$h^{*}_{S,\omega }$
120	77.9%	61.9%	79.2%	79.3%	67.3%	80.3%	80.4%	63.6%	81.5%
238	80.2%	70.2%	81.3%	80.2%	67.8%	81.9%	84.0%	71.4%	86.0%

## Conclusion and future work

In this work, we presented a geometric classification scheme from shape data employing a flexible, yet descriptive characterization of shape variability. Based on a graph convolutional neural network, we further perform transductive inference taking into account the irregular structure in sampling patterns of clinical data sets. Furthermore, we extended the functional characterization of shape variation via an alternative metric that is sensitive to extrinsic curvature and employed a geometric linearization based on the Log-Euclidean framework for positive matrices.

In application to Alzheimer’s disease diagnosis, we achieved classification performance that surpasses recent work for deep learning on 3D surfaces [17] as well as inductive inference from functional descriptions [16]. Furthermore, our method is able to significantly decrease the gap towards Riemannian shape spaces that rely on the more restrictive setting of dense vertex correspondence [32] and for which $80.4\%$ classification accuracy has been reported. Note that Alzheimer’s disease is characterized by various structural and functional changes in the brain implying that diagnosis should be based on a holistic assessment. Indeed, state-of-the-art approaches in Alzheimer’s disease diagnosis are typically based on multiple neuroimaging modalities [38], viz. structural and functional magnetic resonance imaging (s/fMRI), diffusion tensor imaging and positron emission tomography. As the focus of our work is on classification from generalized shape representations, we designed the experiments to explore the descriptive power of the proposed shape representation in combination with GCN-based transductive learning. Nonetheless, the experiments show that our approach achieves classification performance competitive to recent image-based approaches [39, 40] that are conditioned on full sMRI scans. Consequently, a promising direction for future work is to augment our shape-based approach with functional descriptors such as brain connectomes that also lie in the SPD matrix cone [41].

In this extended version, we explored to what extent the Alzheimer’s disease classification can be improved by taking both left and right hippocampi into account. To this end, we expanded the data set introduced in [32] to include both anatomies on the one hand, and, on the other, we add all baseline shapes for which segmentation masks are available in ADNI-2 including those that show topological artifacts, viz. are of genus 1. Conditioning the proposed model on both hippocampi resulted in significant improvements over classification from either left or right ones.

An interesting direction for future work is the graph construction itself, which should be further analyzed as it poses a structural core element in our setup and potentially is the key property for discriminative tasks such as disease grading. Another direction is to explore the rapidly expanding set of graph convolutional architectures for our transductive learning approach. In particular, recent advancements [19] provide neural models that can take advantage of both the geometry of the input domain as well as the feature space alleviating the need for linearization of the latter. Overall, we further plan to investigate the potential of our approach for topologically-varying and incomplete shape collections in order to broaden the scope of shape analysis methodology and to provide more extensive empirical evidence of its performance in the future.

## Data Availability

This work relies on data from the open-access Alzheimer’s Disease Neuroimaging Initiative (ADNI).

## Footnotes

1 adni.loni.usc.edu
